# 1944. Tolerability and Safety of 20-Valent Pneumococcal Conjugate Vaccine (PCV20) in Infants and Older Children in Global Studies

**DOI:** 10.1093/ofid/ofad500.098

**Published:** 2023-11-27

**Authors:** Laurence Flint, Kathleen McElwee, G Laissa Ouedraogo, Noor Tamimi, Mary J Kline, Kimberly J Center, Allison Thompson, Richard de Solom, Masako Yamaji, James Trammel, Lanyu Lei, Yahong Peng, William C Gruber, Daniel Scott, Wendy Watson

**Affiliations:** Pfizer Inc, Pearl River, New York; Pfizer Inc, Pearl River, New York; Pfizer Inc, Pearl River, New York; Pfizer Inc, Pearl River, New York; Pfizer Inc, Pearl River, New York; Pfizer, Collegeville, Pennsylvania; Pfizer Inc, Pearl River, New York; Pfizer Australia, Sydney, New South Wales, Australia; Pfizer R&D Japan, Tokyo, Tokyo, Japan; Pfizer Inc, Pearl River, New York; Pfizer Inc, Pearl River, New York; Pfizer Inc, Pearl River, New York; Pfizer, Collegeville, Pennsylvania; Pfizer, Collegeville, Pennsylvania; Pfizer Vaccine Research and Development, Collegeville, Pennsylvania

## Abstract

**Background:**

The 20-valent pneumococcal conjugate vaccine (PCV20), developed to expand protection against pneumococcal disease, is approved for adults in several countries and for pediatric use in the United States. Tolerability and safety data from PCV20 pediatric studies are described.

**Methods:**

Five randomized studies in infants (1 Phase 2; 4 Phase 3) assessing the safety of PCV20 relative to 13-valent PCV (PCV13) receiving a 3- or 4-dose series (2 or 3 primary series doses and a toddler dose) were conducted in North America, Europe, South America, and Japan. Study vaccines were given intramuscularly in all studies except in Japan, where a subcutaneous (SQ) route was also evaluated. A single-arm Phase 3 study of 1 PCV20 dose was performed in US children 15 months to 17 years old; children < 5 years old had received ≥ 3 doses of PCV13. All studies assessed local reactions and systemic events within 7 days of vaccination. Adverse events (AEs) were collected through 1 month after the primary series (infant studies) and 1 month after the toddler or single vaccine dose. Serious AEs (SAEs) were collected throughout study participation.

**Results:**

The safety population across 6 studies totaled 6654 participants; 3276 infants and 831 children received PCV20 and 2547 infants received PCV13. Overall, the most common local reaction was injection site pain, except in the infant study in Japan, where redness was most common, particularly in SQ groups. In the infant studies, the most common systemic events were irritability and drowsiness. The most common systemic events in older children were fatigue (≥ 2- to < 5-year-old age group) and muscle pain (≥ 5- to ≤ 17-year-old age group). Fever > 40°C was uncommon (PCV20, ≤ 0.5%; PCV13, ≤ 0.1% after any dose). AE and SAE rates were similar to the PCV13 group in the infant studies and low in the study in children. Events were consistent with illnesses and medical conditions that may occur in pediatric populations.

**Conclusion:**

In 6 studies conducted globally, PCV20 was safe and well tolerated in infants and children through 17 years of age. The PCV20 tolerability and safety profile was similar to PCV13 controls in infant studies and consistent with PCV13 studies in children.

FUNDING BY PFIZER INC

**Disclosures:**

**Laurence Flint, MD**, Pfizer Inc: Employee|Pfizer Inc: Stocks/Bonds **Kathleen McElwee, MD, MDH**, Pfizer Inc: Employee|Pfizer Inc: Stocks/Bonds **G. Laissa Ouedraogo, MD**, Pfizer Inc: Employee|Pfizer Inc: Stocks/Bonds **Noor Tamimi, MD**, Pfizer: Employee|Pfizer: Stocks/Bonds **Mary J. Kline, MD**, Pfizer Inc: Employee|Pfizer Inc: Stocks/Bonds **Kimberly J. Center, M.D**, Pfizer Inc: Employee|Pfizer Inc: Stocks/Bonds **Allison Thompson, MD**, Pfizer Inc: Employee|Pfizer Inc: Stocks/Bonds **Richard de Solom, MBBS**, Pfizer Inc: Employee|Pfizer Inc: Stocks/Bonds **Masako Yamaji, MS**, Pfizer Inc: Employee|Pfizer Inc: Stocks/Bonds **James Trammel, MS**, Pfizer Inc: Employee|Pfizer Inc: Stocks/Bonds **Lanyu Lei, PhD**, Pfizer Inc: Employee|Pfizer Inc: Stocks/Bonds **Yahong Peng, PhD**, Pfizer Inc: Employee|Pfizer Inc: Stocks/Bonds **William C. Gruber, MD**, Pfizer, Inc.: Employee|Pfizer, Inc.: Stocks/Bonds **Daniel Scott, MD**, Pfizer Inc: Employee|Pfizer Inc: Stocks/Bonds **Wendy Watson, MD**, Pfizer Inc: Employee|Pfizer Inc: Stocks/Bonds

